# Lipodystrophy as a Late Effect after Stem Cell Transplantation

**DOI:** 10.3390/jcm10081559

**Published:** 2021-04-08

**Authors:** Daniel Tews, Ansgar Schulz, Christian Denzer, Julia von Schnurbein, Giovanni Ceccarini, Klaus-Michael Debatin, Martin Wabitsch

**Affiliations:** 1 Division of Pediatric Endocrinology and Diabetes, Department of Pediatrics and Adolescent Medicine, Ulm University Medical Center, 89075 Ulm, Germany; daniel.tews@uniklinik-ulm.de (D.T.); christian.denzer@uniklinik-ulm.de (C.D.); julia.vonSchnurbein@uniklinik-ulm.de (J.v.S.); 2 Department of Pediatrics and Adolescent Medicine, Ulm University Medical Center, 89075 Ulm, Germany; ansgar.schulz@uniklinik-ulm.de (A.S.); klaus-michael.debatin@uniklinik-ulm.de (K.-M.D.); 3 Obesity and Lipodystrophy Center at the Endocrine Unit, University Hospital of Pisa, 56126 Pisa, Italy; giovanni.ceccarini@unipi.it

**Keywords:** lipodystrophy, adipose tissue, stem cells

## Abstract

Survivors of childhood cancer are at high risk of developing metabolic diseases in adulthood. Recently, several patients developing partial lipodystrophy following hematopoietic stem cell transplantation (HSCT) have been described. In this review, we summarize the cases described so far and discuss potential underlying mechanisms of the disease. The findings suggest that HSCT-associated lipodystrophies may be seen as a novel form of acquired lipodystrophy.

## 1. Introduction

Although modern treatment strategies in childhood malignancies have tremendously improved patient survival, pediatric cancer patients receiving irradiation and/or chemotherapy in combination with hematopoietic stem cell transplantation (HSCT) are at high risk of developing endocrinological complications [1,2,3,4]. This generally includes growth failure, hypothyroidism, and gonadal dysfunction [2,3,4]. Moreover, metabolic diseases have emerged as a part of late complications in patients who received HSCT, including insulin resistance [5,6,7], diabetes mellitus [5,8,9,10], dyslipidemia [5,7,9], central or abdominal obesity [5,9,11], and fatty liver disease [6,9]. Interestingly, metabolic diseases have been diagnosed in the absence of obesity or high body mass index (BMI) [5,10,11,12] and were found associated with total body irradiation (TBI), which is commonly used within the myeloablative conditioning for HSCT [8,10,11]. 

Within a cohort comparing *n* = 21 pediatric patients with acute lymphoblastic leukemia who had received HSCT to cancer patients without HSCT (*n* = 31) and obese controls (*n* = 30), Wei et al. found that these patients had a higher incidence of insulin resistance and glucose intolerance [13]. Moreover, using dual energy X-ray absorptiometry (DEXA) scan analysis, they also found a very different paradigm shift in body fat distribution characterized by increased central to peripheral adiposity, reduced subcutaneous and increased visceral fat towards higher visceral fat depots in patients with HSCT, resembling a lipodystrophic phenotype [13]. 

Lipodystrophies (LD) are a group of rare diseases affecting the growth or distribution of body fat, either characterized by complete loss of adipose tissue (generalized LD) or loss of distinct adipose tissue depots (partial LD). Depending on their pathogenic mechanism, LDs can be further divided into familial/genetic and acquired LDs (for an overview, see [14]). Thus, LDs comprise a large number of different disease entities, some of them so rare that they have only been described in a handful of cases so far [15]. Therefore, a European registry for patients with lipodystrophy (the ECLip registry, ClinicalTrials.gov (NCT03553420)) has been set up to provide a basis for international cooperation and data acquisition [16].

LDs are often accompanied by metabolic complications, including insulin resistance, hypertriglyceridemia, non-alcoholic fatty liver disease (NAFLD), and metabolic syndrome [14,17]. 

Within the last 15 years, several patients who developed partial LD after HSCT in early life were described, suggesting that this disease might be a distinct entity among the late metabolic sequelae of stem cell transplantation [18,19,20].

As HSCT has been recognized as a cause for lipodystrophy, this disease entity, referred to as “Acquired Partial Lipodystrophy associated with total body irradiation and hematopoietic stem cell transplant” or “HSCT-associated lipodystrophy”, has been included in the ECLip registry to facilitate documentation and further research of this rare condition. In this review, we aim to summarize all cases described so far and to discuss common risk factors as well as potential underlying mechanisms of the disease.

## 2. Clinical Cases

Although metabolic alterations and changes in body fat distribution seem to be a common late effect of bone marrow transplantation (BMT), the appearance of lipodystrophy is less common. Within the last years, several patients were described who developed lipodystrophy after pediatric HSCT.

In 2006, Rooney and Ryan described a female patient who received allogeneic BMT in combination with TBI and cyclophosphamide treatment at the age of 14 due to acute lymphoblastic leukemia (ALL) [21]. One year after treatment, she was diagnosed with sclerodermatous graft-versus-host disease (GVHD), which was successfully treated with prednisolone, cyclosporin, and thalidomide over a total period of two years [21]. Nine years after transplantation, the patient developed partial lipodystrophy, mainly at the legs, forearms, thighs, and buttocks. Partial LD was accompanied by diabetes mellitus, hypertriglyceridemia, and markedly low adiponectin levels. Interestingly, liver function was normal apart from slightly elevated liver transaminases. As this phenotype resembled Dunnigan-type lipodystrophy (familial partial lipodystrophy type 2, FPL2), mutations in the lamin A (LMNA) gene, the underlying genetic cause of FPL2, were excluded by sequencing. As the areas of lipoatrophy corresponded well to the sites of cutaneous GVHD, the authors hypothesized that GVHD or immunosuppressive treatment could be the underlying cause of LD development [21]. 

A similar phenotype of body fat distribution was found in a later report describing five cases of LD who received HSCT in childhood due to leukemia or neuroblastoma [18]. As in the aforementioned case, the five patients developed subcutaneous lipoatrophy in the gluteal regions and the extremities, while fat depots in the cheeks, neck, and abdomen were preserved. Mutations in the LMNA gene were absent in four patients, while in the last patient, no genetic examination was performed. All patients had received either allogeneic (*n* = 4) HSCT or autologous peripheral blood stem cell transplantation (*n* = 1) in combination with TBI and intensive chemotherapy due to metastasis or relapse. As a consequence of HSCT, most (*n* = 4) patients developed GVHD, which was treated with chronic immunosuppressive therapy. Metabolic derangements were evident in all patients including hyperinsulinemia (*n* = 4), diabetes mellitus (*n* = 2), dyslipidemia (*n* = 5), and fatty liver disease (*n* = 5). Additionally, levels of leptin and adiponectin were modestly decreased.

In 2017, one of us reported a case of adult lipodystrophy, which developed after early autologous BMT at the age of 2 years to treat acute myeloblastic leukemia (AML) followed by treatment with TBI and allogeneic BMT [20]. Later, the patient received immunosuppressive treatment after developing GVHD. At the age of 13, the patient was diagnosed with severe dyslipidemia, and in the further course—at the age of 17—with T2DM and fatty liver disease. She presented with subcutaneous lipoatrophy in the limbs and gluteal region, while fat at the cheeks was preserved. 

Another case developed acute as well as chronic GVHD after receiving HSCT at the age of 4 years to treat ALL [22]. At the age of 10 years, she was diagnosed with hyperglycemia. CT imaging revealed abnormal body fat distribution and fatty liver, indicating acquired partial lipodystrophy.

Two more cases with partial LD after allogeneic HSCT/TBI were described in 2019 [23]. The first case developed lipoatrophy in the upper and lower extremities and at the gluteal region at the age of 14 years after receiving HSCT in early childhood. The appearance of scleroderma-like skin suggested the suspicion of GVHD development in the patient. The second patient was diagnosed with ALL at the age of seven years and received HSCT, including TBI conditioning, one year later. Consequently, she developed chronic GVHD, which was treated with immunosuppressants, including prednisolone. At the age of 17 years, she presented with partial lipodystrophy affecting the extremities accompanied by diabetes mellitus, dyslipidemia, and fatty liver. The authors of this study speculated that this form of acquired lipodystrophy is a consequence of GVHD affecting the adipose tissue [23].

From these ten cases, it becomes obvious that these patients share a common disease pattern, including partial lipodystrophy with fat loss in the extremities and preserved fat depots in the face, neck, and abdomen accompanied by metabolic disease. All patients described so far (*n* = 10) had a history of total body irradiation at a young age, and the majority (*n* = 9) received immunosuppressive therapy due to GVHD development. Endocrinopathies were also frequently found including growth hormone deficiency (*n* = 6), hypothyroidism (*n* = 5) and hypogonadism (*n* = 6). 

A cross-sectional study in 2017 [19] including *n* = 65 pediatric patients who underwent HSCT for malignancies or hematological disorders brought further information about the frequency of HSCT-associated LD as well as common risk factors associated with this novel disease entity. Interestingly, almost 10% of the children developed partial LD in adolescence with gluteal lipoatrophy and facial lipohypertrophy. The authors found that compared to patients who did not develop LD, patients with LD were older at diagnosis, had a longer elapsed time following HSCT, had more frequently a history of disease recurrence, and were more likely to have undergone multiple HSCTs. In addition, they had higher blood pressure and exhibited higher levels of low-density lipoprotein-cholesterol and triglycerides, whereas their adiponectin levels were significantly lower.

## 3. Common Disease Pattern in HSCT-Associated LD

### 3.1. Body Fat Distribution

Data from all patients published so far with HSCT-associated LD are listed in Table 1. 

All patients diagnosed with partial HSCT-associated LD display a similar pattern of body fat distribution with reduced subcutaneous fat, especially at the extremities (lipoatrophy), preserved or even enlarged fat depots in cheeks, neck, and abdomen. Increased visceral fat deposition associated with fatty liver disease was reported in all cases, except for the patient reported by Rooney and Ryan [21]. This phenotype is reminiscent of Dunnigan-type lipodystrophy based on mutations in the LMNA gene; however, all patients tested were negative for LMNA mutations, ruling out a potential genetic origin of the disease. Body composition in children and adolescents who received HSCT/TBI has been extensively reviewed recently [26]. Accordingly, remodeling of adipose tissue is an early event after HSCT, and there is also evidence of reduced muscle mass in children and adolescents after HSCT [26].

In a study by Wei et al. (*n* = 21) survivors of childhood ALL treated with HSCT and TBI, changes in body composition measured by DEXA were evident in comparison to children with ALL treated with chemotherapy only and obese controls [13]. Overall, BMI, as well as lean mass, was decreased in HSCT/TBI patients compared to both controls. Regarding body fat distribution, HSCT/TBI patients had higher visceral fat and significantly lower subcutaneous fat compared to both chemotherapy-only and obese controls. Moreover, also the prevalence of an increased visceral-to-subcutaneous fat ratio was higher. This indicates that HSCT in combination with TBI favors a general shift from subcutaneous to visceral body fat distribution. Interestingly, as shown by MRI examinations, the group of HSCT/TBI patients was also characterized by a high amount of intramyocellular fat. Among survivors of childhood ALL treated with HSCT and TBI, patients presenting with an overt lipodystrophic phenotype might thus be a more severely affected subgroup. However, in this study, the HSCT/TBI group also demonstrated reduced lean mass, as indicated by a higher prevalence of low fat-free mass index [13]. This description would resemble “sarcopenic obesity”, a clinical condition characterized by an increase in relative fat mass and reduction in lean mass [13].

### 3.2. Metabolic Alterations

Within the published cases with HSCT-associated LD, the occurrence of hypertension, hypertriglyceridemia, insulin resistance, diabetes, and high serum LDL-cholesterol levels were frequent (see Table 1). These features of the metabolic syndrome are likely to be typical comorbidities of lipodystrophy rather than a direct late consequence of HSCT. Indeed, when comparing the small subgroup of HSCT-transplanted patients who developed lipodystrophy with the majority of patients without LD [19], the frequency of these metabolic derangements was higher in the former. Additionally, adiponectin levels were lower in patients with LD as compared to patients without [19], a finding which is also typical for individuals with both generalized [27] and partial lipodystrophy [28]. Interestingly, survivors of childhood ALL treated with HSCT and TBI had circulating levels of adiponectin even lower than obese controls [13]. 

### 3.3. Endocrinopathies

The endocrine system is frequently affected in patients with post-HSCT-related complications. In patients with HSCT-associated LD, endocrine complications such as growth hormone deficiency, hypothyroidism as well as hypogonadism are frequently observed (Table 1). The main risk factors for the development of endocrinopathies include the use of total body irradiation, age at HSCT, and accompanying pharmacologic therapy (for a review, see [29]). Although endocrinopathies are no direct cause of lipodystrophies [14], such complications might contribute to a worse metabolic outcome seen in HSCT-associated LD. In particular, GHD is associated with insulin resistance in adults [30].

## 4. Treatment Strategies for Metabolic Derangements in HSCT-Associated LD 

The present international guidelines for follow-up of survivors of childhood and adolescent cancer or stem cell transplantation consider the importance of early detection of obesity and associated cardiovascular risk factors. Indications, scope, and frequency of clinical investigations vary among guidelines [31,32,33,34,35]. For example, the Scottish SIGN guidelines recommend annual monitoring of weight, height, and BMI in long-term survivors of childhood cancer [33]; similarly, the Center for International Blood and Marrow Transplant Research (CIBMTR) / European Society for Blood and Marrow Transplantation (EBMT) [34] and US COG guidelines [35] recommend this for long-term survivors after stem cell transplantation. At a minimum, blood pressure should be measured annually in long-term survivors [33,34], and lipid status and fasting glucose or HbA1c should be determined every two years in overweight or obese and every five years in normal-weight survivors of childhood cancer according to Scottish Intercollegiate Guidelines Network (SIGN) recommendations [33], and every five years in “standard risk” stem cell transplant survivors [34]. Increased frequency of serum lipid and glucose homeostasis (fasting glucose or HbA1c) testing is indicated in survivors treated with TBI or abdominal radiation (every two years [35] or mediastinal radiation every three to five years [31]), and at three- to six-month intervals after HCT in high-risk patients treated with corticosteroids, or other immunomodulatory therapies [34]. 

Metabolic disorders developing after BMT are not easy to treat. Generally, lifestyle changes are recommended first, with changes in food choices and an increase in physical activity to influence the underlying insulin resistance [36]. In advanced stages of metabolic disorders such as manifest diabetes mellitus, hypertriglyceridemia, and also arterial hypertension, pharmacological therapy with gradual adjustment is required.

Fat loss in lipodystrophy is often associated with a decrease in leptin production and circulating leptin levels [14]. Leptin deficiency results in hyperphagia and can lead to severe hypertriglyceridemia, fatty liver disease, and diabetes, as well as to several other metabolic and endocrine comorbidities [14]. The use of metreleptin to treat hyperglycemia and hypertriglyceridemia in patients with generalized and partial lipodystrophy has been approved first in Japan [37] and later in the European Union [38] and in the UK [39]. In the United States, the FDA has approved the use of metreleptin in generalized LD only [37].

So far, two cases with HSCT-associated lipodystrophy have been reported who were treated with metreleptin to overcome metabolic disease [24,25]. In the first report, a 28-year old female who developed partial lipoatrophy after receiving allogeneic BMT at the age of four years was treated with metreleptin due to poorly controlled hyperglycemia [24]. The patient showed lipoatrophy at the lower extremities accompanied by an accumulation of visceral adipose tissue and fatty liver disease. Treatment with high-dose anti-diabetic combination treatment did not sufficiently improve hyperglycemia in the patient. The authors reported that upon daily administration of metreleptin, the metabolic profile of the patient returned to normal levels. However, the exact treatment regimen and metabolic data after treatment were not presented in this case report. 

In the second study, a 17-year old woman with HSCT-associated LD who developed diabetes, dyslipidemia, fatty liver, and marked insulin resistance was treated with metreleptin for a period of 28 months [25]. Within the treatment period, her blood glucose and lipid parameters, as well as liver function, improved, while there was no significant change in physical activity or food intake. 

Thus, the data from the aforementioned studies demonstrate that metreleptin is a useful drug for the treatment of metabolic disturbances in HSCT-associated LD and further supports that metabolic disease upon HSCT is based on adipose tissue dysfunction.

## 5. Potential Risk Factors for the Development of LD after HSCT

### 5.1. Total Body Irradiation

Total body irradiation is an important component of myeloablative conditioning in ALL treatment regimens. In the studies described so far, all patients with HSCT-associated LD received TBI with a dose of 10 Gy or higher (Table 1), suggesting a causative role of TBI in the disease. Earlier studies have shown that TBI is associated with insulin resistance [5,6,7], diabetes [5,8,9,10], central obesity [5,9,11], dyslipidemia [5,7,9], and fatty liver disease [6,9], but LD is rarely reported upon HSCT. Compared to patients receiving chemotherapy only, the rate of metabolic complications in subjects with TBI/HCST was significantly increased, suggesting TBI as a major determinant of metabolic deterioration [13].

It was suggested earlier that TBI not only affects bone marrow cells but is also likely to damage adipose tissue by depleting precursors within the tissue. Adipocytes differentiate from mesenchymal progenitor cells residing in the adipose tissue vasculature [40]. It has been shown that approximately 10% of the adipocytes are renewed each year by differentiation from preadipocytes [41]. Thus, deterioration of this process may lead to reduced adipose tissue expansion. Indeed, we could recently show in an in vitro study using human preadipocytes that irradiation inhibits preadipocyte proliferation [42]. In vivo, irradiation with sub-lethal (7 Gy) and lethal (10 Gy) doses inhibits proliferation in murine adipose tissues as well and strikingly affects adipose tissue morphology [43], suggesting that the adipose tissue is highly sensitive to radiation exposure. Leptin deficient *ob/ob* mice receiving either syngeneic (from *ob/ob* mice) or congenic (from wild-type C57B6 mice) bone marrow transplants after TBI showed reduced fat accumulation in subcutaneous adipose tissue depots and developed hepatomegaly as well as insulin resistance [44], suggesting that TBI in combination with BMT inhibits adipose tissue expansion leading to metabolic disturbances. 

### 5.2. Graft-versus-Host Disease

One major complication of allogeneic HSCT is the development of graft-versus-host disease (GVHD), which occurs when immunocompetent T-cells in the donated cell preparation (graft) recognize recipient tissue (host) as foreign. In acute GVHD, donor T-cells attack the host tissues, which leads to inflammatory responses in the affected tissues, most commonly the skin, gastrointestinal tract, and liver (for a review, see [45]). Chronic GVHD is a more complex disease that typically affects the skin and lungs [46], but also other organs, including the central nervous system and skeletal muscle [47]. The involvement of B-cell response and autoantibodies in chronic GVHD are reminiscent of autoimmune disease [48]. In most of the cases (*n* = 10/12, see Table 1) with HSCT-associated LD, the development of GVHD was reported as a result of allogeneic HSCT. Although GVHD in the adipose tissue has not been described so far, acquired forms of lipodystrophy display a clear connection between the loss of adipose tissue and autoimmune reactions [49,50,51]. Acquired partial lipodystrophy (APL, Barraquer–Simons syndrome), characterized by lipoatrophy in the face and the upper body segments, is quite often associated with autoimmune disease, suggesting that the pathogenic mechanism may be an expression of autoimmunity [52]. In acquired generalized lipodystrophy (AGL), 25% of the cases are of autoimmune origin, and in another 25%, the onset of AGL is heralded by an episode of lobular panniculitis. The pathogenesis of AGL can vary, and the precise mechanism underlying the fat loss is not resolved precisely [14]. In the rare condition of lipophagic panniculitis, inflammation of the adipose tissue is followed by focal dermal loss of adipocytes, presumably by a cell-mediated autoimmune process [50]. As discussed earlier, fat loss in a patient with AGL was based on CD95-mediated apoptosis of adipocytes [53]. Recently, autoantibodies against perilipin A, a marker of adipocyte lipid droplets, have been detected in patients with AGL [51]. 

Similarities in tissue destruction between GVHD and autoimmune disease, despite different sources of immunoreactive cells, point to a causal role of GVHD in HSCT-associated LD, as suggested earlier [23]. Further research is needed to decipher the presence of GVHD in the adipose tissue and its impact on the development of this rare form of acquired lipodystrophy.

### 5.3. Glucocorticoid Treatment

Besides TBI, the use of chemotherapeutics in myeloablative conditioning, as well as treatment of graft-versus-host disease, may contribute to late HSCT-associated diseases. High-dose glucocorticoids (e.g., prednisolone and dexamethasone) are commonly used in ALL therapy and in GVHD therapy as well. The majority of patients (*n* = 8/11) with HSCT-associated LD received glucocorticoids as GVHD therapy.

The markedly increased prevalence of obesity in survivors of childhood and adolescent ALL, despite the omission of cranial irradiation in most current treatment protocols for standard- and intermediate-risk ALL [54,55], and particularly the characteristic pattern of weight gain in ALL patients during specific phases of therapy [56,57], justify interest in glucocorticoids as a risk factor for the development of obesity. High-dose glucocorticoids are an essential component of induction chemotherapy for ALL (prednisolone, dexamethasone). Glucocorticoid therapy causes full-blown iatrogenic Cushing’s syndrome in pediatric ALL patients, with rapid weight gain, markedly increased energy intake [58], abdominal obesity, and typical other changes in body fat topography. Satiety regulation is disturbed, marked mood and sleep disturbances occur, as does, in a subset of patients, steroid-induced diabetes mellitus. However, studies on the effects of glucocorticoids on obesity risk in cancer survivors show inconsistent results (discussed in [59]), which may be due in part to heterogeneous clinical cohorts and the use of potentially inappropriate anthropometric parameters to reliably detect increased body fat percentage or even sarcopenic obesity. A more recent study from the St. Jude Lifetime Cohort, including *n* = 1996 childhood cancer survivors treated for a wide range of pediatric cancer diagnoses, showed an odds ratio of 1.37 for a BMI ≥ 30 kg/m^2^ after high-dose glucocorticoid exposure over a 25-year observation period [60]. Of note, the obesity-promoting effect of glucocorticoid exposure remained significant after adjustment for other factors also positively associated with obesity risk, including exposure to CRT, younger age at diagnosis, older age at follow-up, obesity at diagnosis, and a number of genetic risk factors identified in a genome-wide single nucleotide polymorphism (SNP) array analysis.

The effect of glucocorticoids on adipose tissue is complex [61]. Adipocytes express both types of glucocorticoid receptors, and glucocorticoids have been shown to promote the differentiation of human adipocyte precursor cells [62]. Data from rodent studies indicate that glucocorticoids enhance adipogenesis but are not a prerequisite for adipose tissue formation [63].

Some of the patients with HSCT-associated LD are reminiscent of a Cushing phenotype [18], suggesting that glucocorticoid treatment may have contributed to the phenotype of the patients. However, excessive weight gain upon treatment has not been reported in any of the patients, questioning the use of glucocorticoids as causative of LD as a late effect of HSCT. 

### 5.4. Transplantation

Patients with HSCT-associated LD have received different types of transplants as a therapy of the primary disease [18,19,20,21,24], including bone marrow HSCT, peripheral blood stem cells (PBSC), as well as cord blood stem cells. Except for one patient who received autologous PBSCs and presented with a moderate phenotype only [18], all other patients underwent allogeneic transplantation. As demonstrated before, lipodystrophy after HSCT was associated with the time elapsed following HSCT, and the receipt of more than one HSCT was higher in patients with LD compared to those who did not develop LD [19]. This suggests that a longer period is required to develop a lipodystrophic phenotype. We thus hypothesize that, upon engraftment of donor HSCTs, and after impairment of adipose tissue expansion due to TBI, the adipose tissue might be gradually replaced by cells from the donor, leading to alterations in adipose tissue growth. This hypothesis is supported by studies demonstrating that a considerable percentage of adipocytes are donor-derived in patients receiving HCST [64,65].

Adipose tissue mostly consists of lipid-laden adipocytes, which account for up to 83% of the adipose tissue volume, but only account for 20%–40% of the total cell number within the tissue [66]. Approximately 10% of the adipocytes are renewed each year by controlled cell death and differentiation from tissue-resident progenitor cells [41]. However, the origin of these progenitor cells is not precisely characterized, and there is no clear surface marker pattern known which could be attributed to adipocyte precursors, although different marker panels have been suggested [67]. Multipotent stem cells that give rise to adipocytes are located in the vascular periphery [40], and it has been demonstrated that different subtypes of adipocyte precursors exist that differentiate into adipocytes with divergent metabolic and endocrine capacities [68]. Murine data also indicate that different subsets of adipocyte progenitors are relevant during development, adulthood, and obesity [69,70]. 

There is growing evidence that adipocyte progenitor cells derived from bone marrow stem cells that migrate into the AT and differentiate into adipocytes. Bone marrow-derived cells represent a heterogeneous cell population that contains different cell types, including hematopoietic stem cells (HSCs), but also less frequently non-hematopoietic mesenchymal stem cells (MSCs) [71]. Besides engraftment of HSC in the bone marrow and replacing the hematopoietic system of the recipient, donor-derived cells have been frequently detected in the epithelium [72,73]. Suggested underlying mechanisms of this tissue chimerism include transdifferentiation of hematopoietic cells, generation of epithelial cells from unknown epithelial precursors and/or universal stem cells in the graft, the fusion of donor hematopoietic cells with recipient epithelial cells, and horizontal gene transfer [74]. Moreover, donor-derived cells have also been found in other tissues, including the liver [75], intestine [76], and adipose tissue [64,65]. 

Several studies using green fluorescent protein (GFP)-labeled bone marrow (BM) transplant mouse models have investigated whether BM cells are able to give rise to adipocytes, leading to diverging results [77,78,79]. Using fluorescence-activated cell sorting (FACS) of BM-derived cells and transplanting them into recipient mice, Majka et al. demonstrated that adipocytes could be differentiated from hematopoietic cells of the BM [78]. Moreover, they also showed that a subpopulation of tissue-resident adipocyte progenitor cells was derived from the myeloid lineage [78]. Interestingly, they also found that BMP-derived adipocytes accumulated over time preferentially in female animals and in the visceral versus the subcutaneous adipose tissue [78].

Recently, two studies independently demonstrated that bone marrow contributes to adipocyte generation also in humans [64,65]. Ryden et al. investigated in *n* = 65 patients who had received allogeneic transplantation of either bone marrow or peripheral blood stem cells (PBSCs) and could detect donor cell engraft in the adipose tissue of all recipients [64]. By the purification of adipocytes and exclusion of leukocyte contaminations, they could demonstrate that a significant percentage of adipocytes (0.1–27%) was donor-derived. The relative number of donor-derived adipocytes was independent of gender, age, and transplantation-related differences but correlated positively with the time since transplantation, suggesting that BM-derived cells contribute to the generation of adipocytes over the entire lifespan. Using mathematical modeling, they calculated that the average contribution of BM cells to adipogenesis was approximately 10%; however, there was large individual variation ranging from 0.2% to 41% [64]. 

Similar data came from a study investigating whether *de novo* generation of adipocytes occurs in a cohort of patients (*n* = 8) who underwent transplantation with allogeneic BM HSCs, mobilized peripheral blood stem cells (PBSCs), or cord blood HSC transplants [65]. In flow-sorted adipocytes, they could identify donor-derived DNA in seven/eight patients, indicating that these cells were generated from transplanted hematopoietic progenitor cells. In consecutive biopsies, they further found an increase in adipocyte chimerism, suggesting that donor-derived adipocytes accumulate over time. 

## 6. Potential Mechanism

**Figure 1:**
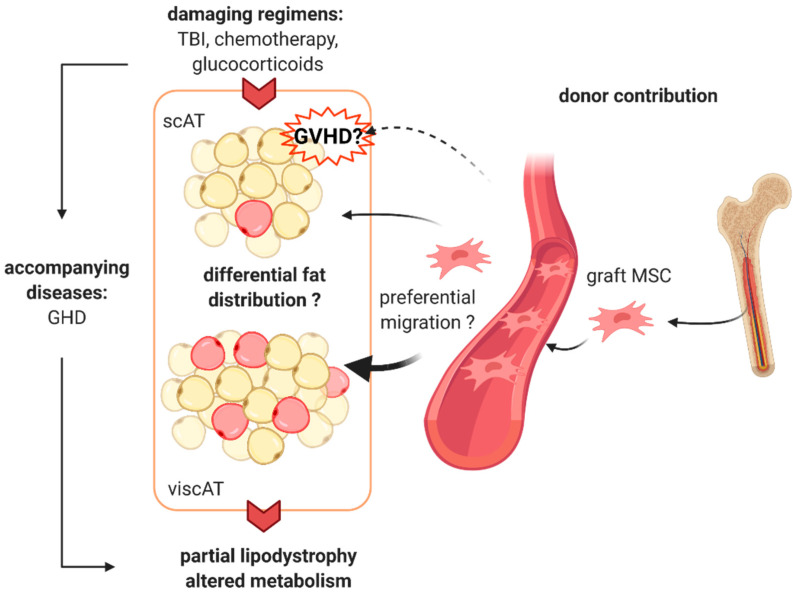
Proposed mechanism of hematopoietic stem cell transplantation (HSCT)-associated lipodystrophy. Total body irradiation (TBI) causes damage to the adipose tissue, most likely by inhibition of preadipocyte expansion and by causing treatment-related endocrine complications, e.g., growth hormone deficiency (GDH). Pharmacologic treatment during myeloablative conditioning and the use of glucocorticoids may further accelerate the disease. Graft versus host disease (GVHD) may be involved in fat loss at the subcutaneous adipose tissue (scAT) level. Donor-derived mesenchymal stem cells (MSC) migrate into the host adipose tissue, where they differentiate into adipocytes and gradually replace the host cells. Preferential migration to the visceral adipose tissue (viscAT) might further contribute to the altered body fat distribution as seen in partial lipodystrophy. The image was created with BioRender.com (created 31 March 2021).

The molecular mechanisms of pathogenesis in HSCT-associated LD are not well understood. From the above-mentioned risk factors potentially causing the development of HSCT-associated lipodystrophies, we would like to expand the current view on the underlying mechanisms [25] by the hypothesis that transplanted donor cells contribute to adipose tissue mass and function (Figure 1). The major prerequisite for the development of the disease seems to be a damaging effect of TBI on adipose tissue. Although not shown directly in vivo in affected patients, the inhibition of adipocyte progenitor cell expansion by radiation seems to be a major contributor to this damage. Pharmacologic treatment during conditioning and GVHD treatment and GVHD itself may further accelerate the disease, as the use of glucocorticoids is associated with similar phenotypes as seen in the patient cohorts. Lipodystrophy often occurs more than 10 years after therapy, indicative of long-term development for the disease. It has been shown that donor-derived adipocytes accumulate over time in the adipose tissue [64,65], indicating that the BM is a reservoir of adipocyte progenitor cells. Replacing patient adipocytes with donor BM-derived cells might have a detrimental impact on adipose tissue homeostasis and metabolism. Higher infiltration rates of BM-derived adipocyte progenitor cells into visceral compared to subcutaneous adipose tissue have been shown in mice [65]. Although not shown in humans so far, this could also contribute to the alteration in body fat distribution. The functional properties of donor-derived adipocytes have not been investigated so far. Thus, further studies are warranted to investigate if donor-derived adipocytes are commonly detected in patients with HSCT-associated LD if tissue-preferential infiltration exists, and if donor-derived adipocytes are metabolically divergent from host adipocytes. 

Taken together, we have reviewed the novel disease entity of HSCT-associated lipodystrophy and describe possible underlying mechanisms in the pathology of the disease. As this is an extremely rare disorder, one has to take into consideration that the disease is often overlooked upon patient follow-up. Implementation of the disease into registries such as the ECLip database will facilitate documentation and further research of this rare condition. 

## Figures and Tables

**Table 1:** Clinical characteristics of patients with lipodystrophies (LD) upon hematopoietic stem cell (HSC) transplantation.

	Ceccarini et al. 2017 [20]	Rooney and Ryan 2006 [21]	Adachi et al. 2013 [18]					Kimura et al. 2017 [22]	Shibata et al. 2018 [24]	Hosokawa et al. 2019 [23]		Adachi et al. 2020 [25]
**Sex**	f	f	f	f	m	m	f	f	F	f	f	f
**Primary diagnosis**	AML	ALL	AML	AML	ALL	NB	NB	ALL	AML	AML	ALL	AML
**Graft**	allogeneic	allogeneic	allogeneic	unrelated BMT	allogeneic	Scheduled PBSCT	allogeneic	allogeneic	allogeneic	Allogeneic	allogeneic	allogeneic
**Age at transplant**	2	14	1	8	0	1	1	4	4	2	7	1
**Irradiation**	TBI	TBI	TBI	TBI	TBI	TBI	TBI	TBI		TBI	TBI	TBI
**Age at diagnosis**	20	23	17	15	19	19	17	10	28	14	17	17
**Treatment-related complications**	GVHD, GH deficiency, hypothyroidism, and hypogonadism.	GVHD.	GVHD, GH deficiency, hypothyroidism, leukoencephalopathy, epilepsy, and hypogonadism.	GVHD, femoral neck necrosis, aplastic anemia, hepatic angioma, hypothyroidism, and primary hypogonadism.	GVHD, GH deficiency, and chronic thyroiditis.	No GVHD, hypothyroidism, empty sella, GH deficiency, and hypogonadism.	GVHD, GH deficiency, hypogonadism, high-frequency deafness, and cataracts.	GVHD.		GVHD.	GVHD.	GVHD, leukoencephalopathy, intractable epilepsy, moderate intellectual impairment, cataracts, GH deficiency, hypothyroidism, and hypogonadism.
**Metabolic complications**	T2DM fatty liver	T2DM low serum AdipoQ	DM Dyslipidemia fatty liver	Hyperinsulinemia dyslipidemia	hyperinsulinemia dyslipidemia	fatty liver increased visceral fat hyperinsulinemia	fatty liver	Hyperglycemia	T2DM fatty liver	Hyperglycemia dyslipidemia	T2DM dyslipidemia	Hyperinsulinemia hypertriglyceridemia fatty liver
**BMI**	14	x	17.7	12.2	16.5	18.3	14.1	x	16.9	13.2	17.0	x
**HbA1c (%)** **(4–5.9)**	7.46	10.5	6.10	5.40	5.30	5.70	5.20	9.2	8.7	6.3	7.3	9.50
**fTG (mg/dL)** **(<100)**	654	1301	675	965	901	1073	402	3090	986	332	927	490
**total cholesterol (mg/dL)**	277	228	322	375	284	314	203	x	x	x	336	x
**HDL cholesterol (mg/dL)**	x	39	39	50	44	44	35	x	40	33	34	x
**LDL cholesterol (mg/dL)**	x		168	203	179	176	124	x	x	x	x	x
**fInsulin (µU/dL)**	x	115.1	x	x	x	x	x	x	x	54	53.9	53.9
**fBG (mg/dL)**	126		x	x	x	x	x	354	232	128	132	100
**LEP (ng/mL)**	7.4	10.60	18.70	9.50	10.70	17.90	11.90	x	6.5	5.6	3.5	x
**AdipoQ (µg/mL)**	x	0.90	1.60	6.80	8.50	1.70	3.80	x	x	1.6	<1.9	1.80
**ALT (U/L)**	62	90	56	102	85	137	250	x	19	40	78	110
**AST (U/L)**	141	x	x	x	x	x	x	x	21	49	85	x
**GGT (U/L)**	140	91	387	x	x	x	x	x	32	x	x	177

f: female; m: male; AML: acute myeloblastic leukemia; ALL: acute lymphoblastic leukemia; BMT: bone marrow transplantation; TBI: total body irradiation; GVHD: graft versus host disease; GH: growth hormone: T2DM; type-2 diabetes miletus; BMI: body mass index; HDL: high-density lipoprotein; LDL: low-density lipoprotein; NB: neuroblastoma; PBSC: peripheral blood stem cell transplant; AdipoQ: Adiponectin; fInsulin: free insulin; fBG: fasting blood glucose; ALT: alanine transaminase; AST: aspartate transaminase; GGT: gamma-glutamyl transferase; LEP: leptin; x: no information given.
